# Disparities in the protagonism of oral health teams in the work process of Primary Healthcare

**DOI:** 10.11606/s1518-8787.2024058005759

**Published:** 2024-04-11

**Authors:** Érika Talita Silva, Raquel Conceição Ferreira, Fabiano Costa Diniz, Milena Ribeiro Gomes, Andréa Maria Eleutério de Barros Lima Martins, Loliza Luiz Figueiredo Houri Chalub, Maria Inês Barreiros Senna

**Affiliations:** I Universidade Federal de Minas Gerais Faculdade de Odontologia Belo Horizonte MG Brasil Universidade Federal de Minas Gerais. Faculdade de Odontologia. Belo Horizonte, MG, Brasil.; II Universidade Federal de Minas Gerais Faculdade de Odontologia Departamento de Odontologia Social e Preventiva Belo Horizonte MG Brasil Universidade Federal de Minas Gerais. Faculdade de Odontologia. Departamento de Odontologia Social e Preventiva. Belo Horizonte, MG, Brasil; III Universidade Estadual de Montes Claros Faculdade de Odontologia Montes Claros MG Brasil Universidade Estadual de Montes Claros. Faculdade de Odontologia. Montes Claros, MG, Brasil; IV Universidade Federal de Minas Gerais Faculdade de Odontologia Departamento de Clínica, Patologia e Cirurgia Odontológicas Belo Horizonte MG Brasil Universidade Federal de Minas Gerais. Faculdade de Odontologia. Departamento de Clínica, Patologia e Cirurgia Odontológicas. Belo Horizonte, MG, Brasil

**Keywords:** Primary Healthcare, Oral Health, Workflow, Outcome Assessment, Health Care, Health Management

## Abstract

**OBJECTIVE:**

Evaluate and compare the protagonism of Oral Health teams (OHt) in the teamwork process in Primary Healthcare (PHC) over five years and estimate the magnitude of disparities between Brazilian macro-regions.

**METHODS:**

Ecological study that used secondary data extracted from the *Sistema de Informação em Saúde para a Atenção Básica* (SISAB – Health Information System for Primary Healthcare) from 2018 to 2022. Indicators were selected from a previously validated evaluative matrix, calculated from records in the Collective Activity Form on the degree of OHt’s protagonism in team meetings and its degree of organization concerning the meeting agendas. A descriptive and amplitude analysis of the indicators’ variation over time was carried out, and the disparity index was also calculated to estimate and compare the magnitude of differences between macro-regions in 2022.

**RESULTS:**

In Brazil, between 3.06% and 4.04% of team meetings were led by OHt professionals. The Northeast and South regions had the highest (3.71% to 4.88%) and lowest proportions (1.21% to 2.48%), respectively. From 2018 to 2022, there was a reduction in the indicator of the “degree of protagonism of the OHt” in Brazil and macro-regions. The most frequent topics in meetings under OHt’s responsibility were the work process (54.71% to 70.64%) and diagnosis and monitoring of the territory (33.49% to 54.48%). The most significant disparities between regions were observed for the indicator “degree of organization of the OHt concerning case discussion and singular therapeutic projects”.

**CONCLUSIONS:**

The protagonism of the OHt in the teamwork process in PHC is incipient and presents regional disparities, which challenges managers and OHt to break isolation and lack of integration, aiming to offer comprehensive and quality healthcare to the user of the Unified Health System (SUS).

## INTRODUCTION

Primary Healthcare (PHC) is configured as the articulating center for users’ access to the Brazilian Unified Health System (SUS). The National Primary Healthcare Policy defines the Family Health Strategy (FHS) as a priority care model for consolidating and expanding PHC coverage in the country^1^ and the strategic space for qualifying the provision of SUS health actions^2^. The work experience at the FHS enables the development of actions aimed at changes in health practice and the autonomy of the subjects participating in this proposal^3^.

The joint, collaborative action of healthcare professionals is one of the pillars of the organization of the work process proposed by the FHS to solve health problems. Linking the Oral Health team (OHt) to the FHS favors reorientating the work process towards a healthcare model^4^, advocating the articulation of actions, the communicative interaction of workers, and overcoming the isolation of knowledge^5^. Oral Health now requires the configuration of a team that interacts with users and other professionals, as well as participates in the management of services to respond to the demands of the population through the planning of individual and collective promotion actions, prevention, and health recovery in a given territory^4^. Carrying out joint activities in Basic Healthcare Units (BHU) and the territory constitutes an essential strategy for teamwork, which can indicate the level of integration between professionals.

The challenges for the organization of oral health work processes in PHC are persistent, such as the weakness in integrating the OHt with the FHS team and the lack of participatory management, which causes dissatisfaction among professionals and users^6^. The OHts develop a few coordinated actions with the other teams in the FHS, with this integration being considered incipient^7,8,9,10^, and improvements in the participation of the OHt in the joint planning of the actions developed^11,12,7,8^ are recommended. Knowing how the OHt work process is being developed can point out advantages and difficulties, directing the planning of actions^13^ aimed at comprehensive and quality care^14^. For this reason, monitoring and evaluation of oral health management and care must be promoted in Brazil to expand the evaluation capacity of public health services.

Monitoring indicators from data obtained in daily health services is essential for evaluating the health work process. The *Sistema de Informação em Saúde para a Atenção Básica* (SISAB – Health Information System for Primary Healthcare) makes data recorded by professionals available through the e-SUS PHC strategy, including collective activities carried out with the population and the team. These activities include meetings between teams and those with local social control bodies^15^. Participation in team meetings is a common responsibility of professionals working in PHC, characterized by joint discussion, planning, and evaluation of their actions based on available data^1^. These meetings favor the comprehensiveness of healthcare actions, contributing to the service’s organization and a better understanding of the needs of the enrolled population^1^. Meetings between users, professionals, and managers promote greater proximity between these social actors and serve as privileged spaces for exercising participatory democracy and social control over healthcare services^16^. From this perspective, six indicators were constructed and validated to measure, in an unprecedented way, OHt’s protagonism in the teamwork process based on data made available by SISAB^2^.

Considering the evidence of regional inequalities in the organization of the OHt work process in PHC^14^, a comparison between Brazilian macro-regions is justified to reveal aspects of OHt’s performance and protagonism in the daily multidisciplinary work in PHC^2^. The analysis that considers regional contexts can support decision-making, having as a reference the principle of equity, with a commitment to comprehensive care, and the qualification of attention to users, valuable and opportune for the new cycle of Oral Health Policies, started in 2023^2^. In this context, the objective of this study was to evaluate and compare OHt’s protagonism in the teamwork process in PHC over five years (from 2018 to 2022) and estimate the magnitude of disparities between Brazilian regions for 2022.

## METHOD

This nationwide ecological study used public secondary data extracted from SISAB in January 2023. The indicators analyzed were: degree of OHt’s protagonism in team meetings (IND1); degree of OHt’s organization concerning the teamwork process (IND2); degree of OHt’s organization concerning administrative/operational issues (IND3); degree of OHt’s organization concerning the diagnosis and monitoring of the territory (IND4); degree of OHt’s organization concerning case discussion and singular therapeutic project (IND5); and degree of OHt’s organization concerning permanent education (IND6). A committee of judges validated these indicators, and their measurability was tested using data from 2020^2^. They form the subdimension “Oral Health Team Work Process” of the Oral Health Management dimension, of the Monitoring Indicator Matrix and Assessment of the Quality of Oral Health Services^2^.

The data used to calculate the indicators are generated from registration in the Collective Activity Form in the Simplified Data Collection (CDS) applications online or offline, in the e-SUS PHC Collective Activity application (on Android^®^ devices), or via proprietary systems that feed SISAB. The variables used to obtain the indicators refer to collective actions for team organization, which include team meetings, meetings with other healthcare teams, and intersectoral meetings/local health council social control. Meeting topics may be one or more of the following: administrative/operational issues; work process; territory diagnosis/territory monitoring; team action planning/monitoring; and/or discussion of a unique therapeutic case/project and permanent education. The professional’s data, the *Cartão Nacional de Saúde* (CNS – National Health Card), and the *Classificação Brasileira de Ocupações* (CBO – Brazilian Classification of Occupations), mediators of collective activity, must be informed in each record and are mandatory^15^.

Data on the numerator and denominator of each indicator was obtained by consulting the SISAB “*Relatório de Atividade Coletiva na Atenção Básica*” (Collective Activity Report in Primary Healthcare), which was extracted at the national level and for the five Brazilian macro-regions for each year between 2018 and 2022.

The degree of OHt’s protagonism (IND1) refers to the proportion of meetings under the responsibility of OHt professionals. It is calculated by the ratio between the number of team meetings, meetings with other healthcare teams, intersectoral/local health council/ Social Control meetings under the responsibility of an OHt professional in a given location and period, and the number of meetings held in the same location and period^2^. The other teams are the Family Health Team (FHt), Community Health Agent Team, Family Health Support Center, Basic Healthcare Team, Street Clinic Team, Prison Basic Healthcare Team; and Primary Healthcare Team^15^.

The other indicators measure the proportion of themes recorded in meetings under the responsibility of OHt professionals, estimated by the ratio between the number of team meetings, meetings with other healthcare teams in which the responsible professional was a member of the OHt with one of the topics registered (administrative/operational issues, work process, territory diagnosis/territory monitoring, team action planning/monitoring, case discussion/single therapeutic project, and permanent education) in a given location and period; and the number of team meetings and meetings with other teams, in which the responsible professional was a member of the OHt regardless of the registered theme, in the same location and period^2^. For data extraction, filters were selected in SISAB according to the data to be captured, following the guidelines in Figure to obtain the numerator (NUM) and denominator (DEM) of each indicator^2^.

**Figure:**
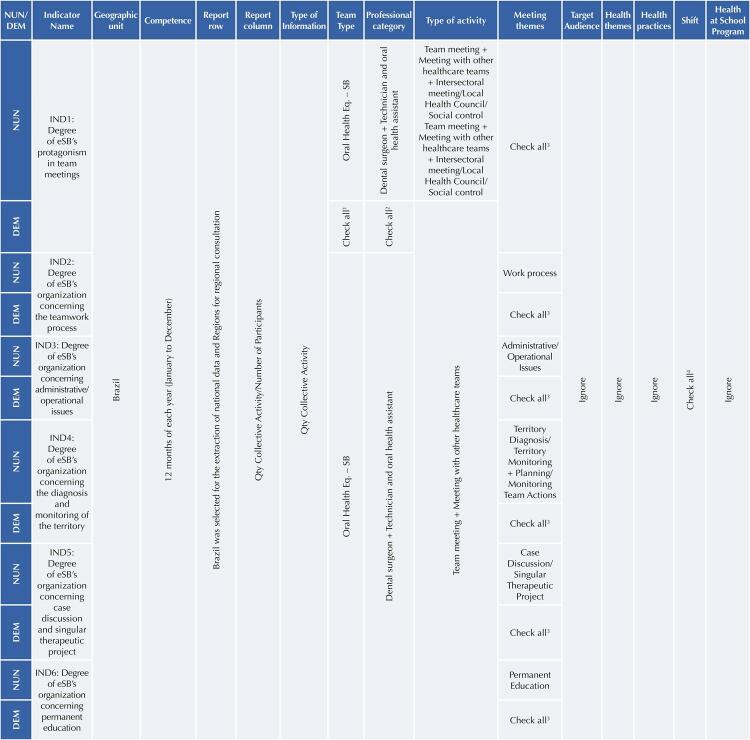
Filters in SISAB to obtain the numerator (NUN) and denominator (DEM) of indicators2

List of Acronyms: IND: Indicator/NUM: Numerator/DEM: Denominator/Eq: Team/SB: Oral Health

^1^ Other teams: Family Health Team - eSF, Community Health Agent Team - eACS, Family Health Support Center - NASF, Primary Healthcare Team - eAB, Street Clinic Team - eRC, Prison Basic Healthcare Team - eABP and Primary Healthcare Team - eAP.

^2^ Professionals: Community health agent, Endemic disease control agent, Health agent, Social worker, Dental surgeon, Social educator, Nurse, Pharmacist, Physiotherapist, Speech therapist, Physician, Veterinarian, Nutritionist, Other high school-level professionals, Other higher education professionals, Physical education professional, Psychologist, Sanitarian, Nursing technician and assistant, Oral health technician and assistant, Occupational therapist, Naturopath, Music therapist, Art therapist, Holistic therapist, Receptionist.

^3^ Meeting themes: Administrative/operational issues, Work processes, Diagnosis of the territory/monitoring of the territory, Planning/monitoring of team actions, Case discussion/singular therapeutic project, Permanent education, Others (Themes do not fit into any of these themes).

^4^ Shifts: Morning, Afternoon, Night.

The extraction process generated two spreadsheets per indicator in Excel^®^ format: one for the denominator and another for the denominator per year. Then, these bases were linked, considering the common variables that indicated the level of national or each region’s disaggregation, and the indicators were calculated, dividing the numerator by the denominator, and multiplying by 100 to obtain the values in percentages.

The descriptive analysis of the indicators was carried out considering the values calculated for Brazil and disaggregated for each macro-region over time (2018–2022). The annual percentage variation for each indicator was estimated for Brazil and each macro-region by the rate of variation (RV), using the following calculation method: *[(indicator result in the subsequent year ÷ indicator result in the previous year)-1]×100*. The variation in the entire period was estimated by the average of the four variations obtained. The Disparity Index (DI) was used to estimate and compare the magnitude of differences in indicators between Brazilian macro-regions in 2022. The values of this index indicate the average deviation of the proportions observed in a macro-region concerning the reference proportion in percentage, i.e., the spread of proportions around the reference value. In this study, the disparity index was calculated considering the macro-region with the highest proportion of activities carried out. The calculation was based on the approach described by Pearcy and Keppel^17^, using the following formula: *ID* = ∑[|*ri* – *R*|/*n*] * 100/*R*, where *ri =* percentage of the indicator, *R* = reference value, and *n* = number of regions.

## RESULTS

Low proportions of meetings under OHt responsibility were observed over time (Table 1). In Brazil, the values varied between 3.06% and 4.04%. Higher (3.71% to 4.88%) and lower proportions (1.21% to 2.48%) were observed for the Northeast and South regions, respectively, throughout the analyzed period. The lowest proportions of meetings under OHt’s responsibility in Brazil and macro-regions were observed in 2020, coinciding with the beginning of the COVID-19 pandemic. It can also be seen that there was a reduction (57%) in the total number of meetings held by all PHC teams in Brazil, from 1,029,090 in 2019 to 594,760 in 2020 (Table 1). The annual percentage variations in the indicator of the degree of OHt’s protagonism revealed an unstable pattern of change, with an increase and reduction between years and differences between macro-regions in these variations. A reduction of ≥ 19.91% was consistently observed for all Brazilian macro-regions in 2020 compared with 2019. This reduction was noted to be followed by a positive variation rate in all macro-regions in the following period (from 2020 to 2021), remaining stable or with minor negative variations in the last year analyzed (Table 2). The differences in the indicator of the “degree of protagonism of the OHt” between the regions were similar throughout the period, consistently showing lower and higher values in the South and Northeast regions.

**Table 1:** Description of teamwork process indicators. Brazil and Brazilian macro-regions (2018-2022).

Region	2018			2019			2020			2021			2022		
				
	N	D	IND (%)	N	D	IND (%)	N	D	IND (%)	N	D	IND (%)	N	D	IND (%)
IND1 - Degree of OHt’s protagonism in team meetings															
Brazil	35830	886283	4.04	41531	1029090	4.04	18223	594760	3.06	19174	577055	3.32	23070	694807	3.32
Midwest	2522	59647	4.23	2843	67302	4.22	1320	39057	3.38	1467	37461	3.92	1896	49902	3.80
Northeast	19441	410953	4.73	24442	500704	4.88	9907	266898	3.71	9750	246386	3.96	10344	259148	3.99
North	2243	70715	3.17	3450	98437	3.50	1524	64403	2.37	1737	63213	2.75	1859	70801	2.63
Southeast	8738	228408	3.83	8256	252250	3.27	4776	167061	2.86	5438	170508	3.19	7638	230051	3.32
South	2886	116560	2.48	2540	110397	2.30	696	57341	1.21	782	59487	1.31	1333	84905	1.57
IND2 - Degree of OHt’s organization concerning the teamwork process															
Brazil	17842	32613	54.71	21612	37845	57.11	10799	17332	62.31	11132	17843	62.39	13129	21217	61.88
Midwest	1480	2387	62.00	1798	2665	67.47	832	1249	66.61	1000	1416	70.62	1263	1788	70.64
Northeast	9097	17556	51.82	12428	22336	55.64	5980	9499	62.95	5641	9142	61.70	5767	9652	59.75
North	1332	2108	63.19	1924	3170	60.69	969	1460	66.37	1009	1594	63.30	1226	1741	70.42
Southeast	4316	8025	53.78	4136	7519	55.01	2592	4499	57.61	3063	5021	61.00	4122	6861	60.08
South	1617	2537	63.74	1326	2155	61.53	426	625	68.16	413	670	61.64	685	1175	58.30
IND3 - Degree of OHt’s organization concerning administrative/operational issues															
Brazil	13301	32613	40.78	16765	37845	44.30	8051	17332	46.45	8117	17843	45.49	9048	21217	42.65
Midwest	1078	2387	45.16	1438	2665	53.96	762	1249	61.01	811	1416	57.27	1069	1788	59.79
Northeast	6852	17556	39.03	9933	22336	44.47	4630	9499	48.74	4351	9142	47.59	4197	9652	43.48
North	946	2108	44.88	1285	3170	40.54	632	1460	43.29	694	1594	43.54	791	1741	45.43
Southeast	3109	8025	38.74	2941	7519	39.11	1688	4499	37.52	1912	5021	38.08	2413	6861	35.17
South	1316	2537	51.87	1168	2155	54.20	339	625	54.24	349	670	52.09	578	1175	49.19
IND4 - Degree of OHt’s organization concerning the diagnosis and monitoring of the territory															
Brazil	13335	32613	40.89	16332	37845	43.15	7429	17332	42.86	7524	17843	42.17	9469	21217	44.63
Midwest	833	2387	34.90	1151	2665	43.19	479	1249	38.35	571	1416	40.32	820	1788	45.86
Northeast	7394	17556	42.12	10057	22336	45.03	4550	9499	47.90	4294	9142	46.97	4371	9652	45.29
North	849	2108	40.28	1189	3170	37.51	489	1460	33.49	638	1594	40.03	716	1741	41.13
Southeast	3112	8025	38.78	2987	7519	39.73	1615	4499	35.90	1665	5021	33.16	2923	6861	42.60
South	1147	2537	45.21	948	2155	43.99	296	625	47.36	356	670	53.13	639	1175	54.38
IND5 - Degree of OHt’s organization concerning case discussion and singular therapeutic project															
Brazil	3606	32613	11.06	4182	37845	11.05	1694	17332	9.77	1675	17843	9.39	2710	21217	12.77
Midwest	317	2387	13.28	384	2665	14.41	156	1249	12.49	188	1416	13.28	287	1788	16.05
Northeast	1168	17556	6.65	1527	22336	6.84	621	9499	6.54	498	9142	5.45	564	9652	5.84
North	192	2108	9.11	232	3170	7.32	114	1460	7.81	98	1594	6.15	145	1741	8.33
Southeast	1353	8025	16.86	1482	7519	19.71	667	4499	14.83	724	5021	14.42	1486	6861	21.66
South	576	2537	22.70	557	2155	25.85	136	625	21.76	167	670	24.93	228	1175	19.40
IND6 - Degree of OHt’s organization concerning permanent education															
Brazil	6437	32613	19.74	7352	37845	19.43	3501	17332	20.20	3523	17843	19.74	4445	21217	20.95
Midwest	463	2387	19.40	504	2665	18.91	226	1249	18.09	286	1416	20.20	313	1788	17.51
Northeast	2882	17556	16.42	3526	22336	15.79	1395	9499	14.69	1374	9142	15.03	1585	9652	16.42
North	432	2108	20.49	758	3170	23.91	372	1460	25.48	342	1594	21.46	430	1741	24.70
Southeast	2081	8025	25.93	2056	7519	27.34	1355	4499	30.12	1386	5021	27.60	1809	6861	26.37
South	579	2537	22.82	508	2155	23.57	153	625	24.48	135	670	20.15	308	1175	26.21

N: Numerator; D: Denominator; IND: Indicator

**Table 2:** Rate of variation (RV) of teamwork process indicators. Brazil and Brazilian macro-regions (2018-2022) (%)

Brazil/Regions	RV	RV	RV	RV	Average RV
				
	2018–2019	2019–2020	2020–2021	2021–2022	2018–2022
IND1 - Degree of OHt’s protagonism in team meetings					
Brazil	0.00	-24.26	8.50	0.00	-3.94
Midwest	-0.24	-19.91	15.98	-3.06	-1.81
Northeast	3.17	-23.98	6.74	0.76	-3.33
North	10.41	-32.29	16.03	-4.36	-2.55
Southeast	-14.62	-12.54	11.54	4.08	-2.89
South	-7.26	-47.39	8.26	19.85	-6.64
IND2 - Degree of OHt’s organization concerning the teamwork process					
Brazil	4.39	9.11	0.13	-0.82	3.20
Midwest	8.82	-1.27	6.02	0.03	3.40
Northeast	7.37	13.14	-1.99	-3.16	3.84
North	-3.96	9.36	-4.63	11.25	3.01
Southeast	2.29	4.73	5.88	-1.51	2.85
South	-3.47	10.78	-9.57	-5.42	-1.92
IND3 - Degree of OHt’s organization concerning administrative/operational issues					
Brazil	8.63	4.85	-2.07	-6.24	1.29
Midwest	19.49	13.07	-6.13	4.40	7.71
Northeast	13.94	9.60	-2.36	-8.64	3.14
North	-9.67	6.78	0.58	4.34	0.51
Southeast	0.96	-4.07	1.49	-7.64	-2.32
South	4.49	0.07	-3.96	-5.57	-1.24
IND4 - Degree of OHt’s organization concerning the diagnosis and monitoring of the territory					
Brazil	5.53	-0.67	-1.61	5.83	2.27
Midwest	23.75	-11.21	5.14	13.74	7.86
Northeast	6.91	6.37	-1.94	-3.58	1.94
North	-6.88	-10.72	19.53	2.75	1.17
Southeast	2.45	-9.64	-7.63	28.47	3.41
South	-2.70	7.66	12.18	2.35	4.87
IND5 - Degree of OHt’s organization concerning case discussion and singular therapeutic project					
Brazil	-0.09	-11.58	-3.89	36.00	5.11
Midwest	8.51	-13.32	6.33	20.86	5.60
Northeast	2.86	-4.39	-16.67	7.16	-2.76
North	-19.65	6.69	-21.25	35.45	0.31
Southeast	16.90	-24.76	-2.76	50.21	9.90
South	13.88	-15.82	14.57	-22.18	-2.39
IND6 - Degree of OHt’s organization concerning permanent education					
Brazil	-1.57	3.96	-2.28	6.13	1.56
Midwest	-2.53	-4.34	11.66	-13.32	-2.13
Northeast	-3.84	-6.97	2.31	9.25	0.19
North	16.69	6.57	-15.78	15.10	5.65
Southeast	5.44	10.17	-8.37	-4.46	0.70
South	3.29	3.86	-17.69	30.07	4.88

In Brazil, the topics most frequently discussed in team meetings were, throughout the period, work process (54.71% to 70.64%), diagnosis and monitoring of the territory (33.49% to 54.48%), and administrative/operational issues (35.17% to 61.01%). The least frequent topics were permanent education (14.69% to 30.12%) and case discussion/single therapeutic project (5.45% to 25.85%) (Table 1). This distribution was repeated when considering each macro-region separately, with a greater frequency of discussion of the teamwork process theme in all macro-regions.

Table 2 highlights the slight variation in indicators over time in all macro-regions and their differences in the proportions observed. Annual percentage variations were unstable in magnitude between macro-regions and over time. The averages of these variations over the entire period (2018–2022) were positive for most indicators in Brazil and in most macro-regions, signaling a pattern of increasing proportions.

IND4 – “Diagnosis and monitoring of the territory” presented the highest rates of positive variation in 2 moments: 2018-2019 (23.75%) in the Midwest region and 2020-2021 (19.53%) in the North region. IND5 – “Case discussion and singular therapeutic project” presented the most significant negative variations in all periods, in two specific macro-regions, in 2018-2019 (-19.65%) and 2020-2021 (-21.25 %) in the North region and 2019-2020 (-15.82%) and 2021-2022 (-22.18%) in the South region. A significant fluctuation in IND5 was observed in the Southeast region, which showed a positive variation in the period 2018-2019 (16.90%), then a negative variation in 2019-2020 (-24.76%), and again a negative variation in 2020-2021 (-2.76%) and then the most significant variation found for all indicators studied in 2021-2022 (50.21%), resulting in a positive average of (9.90%) for the period 2018-2019.

In 2022, different magnitudes of disparities were observed between regions, with the highest values observed for the indicators IND 5 – “Degree of OHt’s organization concerning case discussion and singular therapeutic project” (DI = 34.2), IND1 – “Degree of OHt’s protagonism in team meetings” (DI = 23.3), and IND3 – “Degree of OHt’s organization concerning administrative/operational issues” (DI = 22.0) (Table 3).

**Table 3:** Disparity Index (DI) between geographic regions in the degree of protagonism and organization of the OHt concerning team meeting themes, Brazil, 2022.

Brazil/Regions	Indicador 2022 (%)	Disparity Index (DI)
IND1 - Degree of OHt’s protagonism in team meetings		
Brazil	3.32	23.3
Midwest	3.80	
Northeast	3.99*	
North	2.63	
Southeast	3.32	
South	1.57	
IND2 - Degree of OHt’s organization concerning the teamwork process		
Brazil	61.88	9.4
Midwest	70.64	
Northeast	59.75	
North	70.42*	
Southeast	60.08	
South	58.30	
IND3 - Degree of OHt’s organization concerning administrative/operational issues		
Brazil	42.65	22
Midwest	59.79*	
Northeast	43.48	
North	45.43	
Southeast	35.17	
South	49.19	
IND4 - Degree of OHt’s organization concerning the diagnosis and monitoring of the territory		
Brazil	44.63	15.7
Midwest	45.86	
Northeast	45.29	
North	41.13	
Southeast	42.60	
South	54.38*	
IND5 - Degree of OHt’s organization concerning case discussion and singular therapeutic project		
Brazil	12.77	34.2
Midwest	16.05	
Northeast	5.84	
North	8.33	
Southeast	21.66*	
IND6 - Degree of OHt’s organization concerning permanent education		
Brazil	20.95	15.7
Midwest	17.51	
Northeast	16.42	
North	24.70	
Southeast	26.37*	
South	26.21	

*Regions with the highest proportion in the results of the indicators, used as reference values for calculating the disparity index.

It is noteworthy that the Northeast region presented the highest value for IND1 – “Degree of OHt’s protagonism in team meetings” and the lowest values for IND 5 – “Degree of OHt’s organization concerning case discussion and singular therapeutic project” and for IND3 – ”Degree of OHt’s organization concerning administrative/operational issues.”

The opposite was observed for the South region, with a lower value for IND1 – “Degree of OHt’s protagonism in team meetings” and higher values for IND3 and IND5. The smallest magnitude of disparity between macro-regions was observed for IND2 – “Degree of OHt’s organization concerning the teamwork process” (DI = 9.4) (Table 3).

## DISCUSSION

This work evaluated, unprecedentedly, OHt’s protagonism in the teamwork process in PHC in Brazil and its macro-regions through indicators prepared from SISAB data. The results showed a small proportion of meetings on OHt responsibility and seemed to indicate the difficulties in co-managing the healthcare work process in PHC. Collaborative practice, communication between teams, and sharing of power aiming to overcome fragmentation, isolated professional performance, and hierarchical work relationships^5^ are challenges faced in healthcare services. Encouraging the protagonism of healthcare workers is one of the principles of SUS’s National Humanization Policy^18^, which aims at the participation of teams in decision-making processes at work, to consolidate the knowledge they build in their daily lives^19^.

Team meetings are spaces for strengthening the protagonism of professionals, considering that their periodic holding is a strategy for bringing together team integration and planning^20^. As meetings are the most used resource to promote communication between professionals and users, as well as to promote teamwork, the low protagonism seems to be a reflection of the professional practices of the OHt still being marked by isolation and little interaction and participation in collective work management and participatory management processes in PHC. On the other hand, they may also reflect structural and interaction problems in holding meetings, such as lack of physical space, current management model and oral healthcare; the existence of conflicting and distant relationships; little availability of time on the part of professionals and little institutional appreciation of this type of activity^5;20^; non-existence and/or low visibility of local health councils in the territories; and also, a small appropriation of these spaces by OHt.

These findings may also be related to the difference in population coverage between OHt and FHt. In 2020, FHt coverage in Brazil was 63.62%, while OHt coverage was 44.95%. Among the macro-regions, differences are observed in the coverage of these teams, with disparities between regions of 82.33% and 69.56% in the Northeast for FHt and OHt, respectively, and 50.99% and 30.09%, respectively in the Southeast^21^. This disproportion can deepen the barriers to their protagonism in meetings.

There was instability in the temporal variation in the participation of the OHt as responsible for the meetings, demonstrating that there was no standard of action for the team. The negative variation observed from 2019 to 2020 in all regions corresponds to the period in which the work process within the scope of PHC, actions, and services was redefined, with a significant reduction in face-to-face collective activities aiming to contain Coronavirus transmission^22^. Advances in oral health work in the FHS require the management to encourage professionals to seek skills and competencies and to have attitudes to propose intervention actions^10^ to promote positive changes in the health service. A study that analyzed the performance of institutional support regarding the participation of the OHt in monitoring actions, team meetings, and organization of the work process in PHC identified that there was a positive association between the actions carried out by the institutional supporter and the non-clinical actions of the OHt in Brazil^23^.

Despite the low proportion of meetings coordinated by the OHt, the themes recorded cover, in different proportions, aspects related to the organization of the health work process in the BHU and the territory. The most frequent themes were teamwork processes and administrative and operational aspects, both the BHU and the territory’s diagnosis and monitoring of actions. The work process theme presented higher proportions in all years of the period studied, with positive averages in the rates of change in all regions except the South region. Furthermore, this theme demonstrated the lowest disparity rate, suggesting a possible trend over time.

The greater frequency of these themes may indicate a greater demand for accomplishment or more consolidated activities in teamwork. On the other hand, by dedicating more time to these themes, teams may fail to discuss other equally essential topics for organizing the work process. The most significant disparities were observed in the indicators related to the theme of case discussion/single therapeutic project (IND5) and in the degree of OHt’s protagonism (IND1). IND5 also showed higher rates of negative variation in the period studied, and IND1 had negative variation in all regions on average for the years of the study (2018–2022). These same indicators also presented the lowest proportions in the values calculated over time.

The themes of case discussion/singular therapeutic project and permanent education were less frequent, indicating that user care may be segmented and directed toward treatments that do not consider therapeutic possibilities in the territory itself^24^. By building the therapeutic project in a shared way with the team, it is possible to find answers to oral health needs in a more decisive way^25^and expand the bond with users^7^.

Permanent Health Education (EPS) is a strategy that seeks to qualify workers, favoring teamwork, participatory management, and co-responsibility in the teaching-learning processes to achieve the strategic objectives of the SUS^26^. The low proportion of meetings with the EPS theme may be related to work overload, the lack of planning to carry out EPS initiatives, and the lack of appreciation of these initiatives by management, among others^29^.

Our findings support that regional differences and the heterogeneity of the OHt work process in Brazil remain^14,30^, indicating that the performance of healthcare services is subordinated to contextual determinants. A nationwide study showed that the Southeast, South, and Northeast regions had the highest frequencies of the OHt with better performance in the work process regarding the use of instruments used for action planning, healthcare promotion actions, and comprehensive care. Action planning also proved challenging, with lower proportions of consolidated OHt types I and II and more significant disparity between Brazilian regions^14^. Identifying and understanding socioeconomic differences and the organization of healthcare services can help managers and professionals act to reduce local-regional disparities in the organization of the OHt work process.

This study estimated indicators aggregated by Brazilian macro-regions with an exploratory and descriptive approach, making it impossible to analyze the variability between municipalities in the same region. This level of disaggregation was chosen, depending on the percentage of municipalities without records of meetings in the period, in all regions, regardless of the responsible professional, corresponding to the IND1 denominator: North (41.5% to 54.4%), Northeast (6.5% to 25.8%), Southeast (22.1% to 39.2%), Midwest (29.1% to 57.4%), and South (0 to 39.7%).

Added to this aspect was the fact that many of the municipalities, in all regions, showed no record of meetings under the responsibility of OHt. In favor of the IND1 analysis, all these cases assumed in the numerator that OHt was not responsible for any meeting in the period analyzed. The percentages of municipalities with no meetings under OHt’s responsibility in the period were: North (39.1% to 16.9%), Northeast (55.7% to 63.2%), Southeast (52.5% to 62.9%), Midwest (39.9% to 61.1%), and South (51.5% to 83.8%).

IND2 to IND6 were analyzed considering the total number of meetings under OHt’s responsibility in each macro-region, registered in municipalities with the presence of at least one in the period, which corresponded to the following percentages: North (6.4 to 16, 2%), Northeast (13.2% to 37.5%), Southeast (6.8% to 16.6%), Midwest (1.8% to 12.3%), and South (6.8% to 24.8%). They signal and reinforce the fragility of the OHt’s protagonism in team meetings and with the local health councils in PHC, showing the need to qualify registration in the e-SUS PHC system to give new meaning and reaffirm collective activities as a central element for the work process in health.

Some studies indicate that investments in technological resources, qualification and training of professionals, and support are necessary to qualify the implementation and use of information systems in the country, especially for the e-SUS PHC strategy^30,31^. While sources of secondary data made available through nationally based information systems, such as SISAB, constitute relevant sources, given their scope and capillarity, the low quality and absence of records continue to be a barrier to their use.

Despite the limitations, this initial exploratory analysis of unpublished indicators demonstrated the national panorama concerning the protagonism and degree of organization of OHts in their teamwork process in Brazil and the macro-regions. Furthermore, the use and evaluation of the quality of SISAB data can contribute to its improvement, consolidating itself as an essential source for studies on the work processes of PHC teams.

Another limitation of this study was the definition of OHt’s protagonism adopted in the construction of the indicator, which considered it only when the professional responsible for the meeting was the dental surgeon, oral health technician, or assistant. This definition may not necessarily correspond to the level of OHt’s protagonism in conducting activities and its effective participation in the daily teamwork process in PHC^2^. Through the calculated indicator, there is also the impossibility of establishing comparisons of the protagonism between different professional categories that work in PHC since a relationship is not established between meetings under the responsibilities of different professionals. In this sense, developing new indicators and future studies are necessary.

Despite these limitations, indicators created based on the daily work of teams in PHC are considered to have great potential for analyzing geographic and temporal variations in certain regions, states, and/or municipalities, observing the results together or separately, and allowing the analysis of disparities in the management of the OHt work process and their possible causes. Investigating these indicators can identify inequality and trends that require specific actions and studies, contributing to decision-making by PHC professionals and managers^2^.

The protagonism of the OHt in the teamwork process in PHC in Brazil is still incipient. It presents disparities between macro-regions, challenging managers, and professionals to overcome isolation and little integration between different categories of workers to pay attention to comprehensive and quality healthcare for SUS users.
