# Supplementary material for: Disparities in the protagonism of oral health teams in the work process of Primary Healthcare
Source: Rev Saude Publica. 2024 Apr 11;58:14. doi: 10.11606/s1518-8787.2024058005759 (PMC11037909; doi:10.11606/s1518-8787.2024058005759)
Supplement: Translation

# Translation: Disparidades do protagonismo das equipes de saúde bucal no processo de trabalho na APS

**Authors:** Érika Talita Silva, Raquel Conceição Ferreira, Fabiano Costa Diniz, Milena Ribeiro Gomes, Andréa Maria Eleutério de Barros Lima Martins, Loliza Luiz Figueiredo Houri Chalub, Maria Inês Barreiros Senna

**Affiliations:** I Brasil Universidade Federal de Minas Gerais. Faculdade de Odontologia. Belo Horizonte, MG, Brasil.; II Brasil Universidade Federal de Minas Gerais. Faculdade de Odontologia. Departamento de Odontologia Social e Preventiva. Belo Horizonte, MG, Brasil; III Brasil Universidade Estadual de Montes Claros. Faculdade de Odontologia. Montes Claros, MG, Brasil; IV Brasil Universidade Federal de Minas Gerais. Faculdade de Odontologia. Departamento de Clínica, Patologia e Cirurgia Odontológicas. Belo Horizonte, MG, Brasil

**Keywords:** Atenção Primária à Saúde, Saúde Bucal, Fluxo de Trabalho, Avaliação de Resultados em Cuidados de Saúde, Gestão em Saúde

## Abstract

**OBJETIVO:**

Avaliar e comparar o protagonismo das equipes de Saúde Bucal (eSB) no processo de trabalho em equipe na Atenção Primária à Saúde (APS) ao longo de cinco anos, e estimar a magnitude das disparidades entre as macrorregiões brasileiras.

**MÉTODOS:**

Estudo ecológico que utilizou dados secundários extraídos do Sistema de Informação em Saúde para a Atenção Básica (SISAB), de 2018 a 2022. Foram selecionados indicadores de matriz avaliativa previamente validada, calculados a partir dos registros na Ficha de Atividade Coletiva do grau de protagonismo das eSB nas reuniões de equipe, bem como do seu grau de organização em relação às pautas dos encontros. Foi realizada análise descritiva e da amplitude da variação dos indicadores ao longo do tempo, e também foi calculado o índice de disparidade para estimar e comparar a magnitude das diferenças entre as macrorregiões no ano de 2022.

**RESULTADOS:**

No Brasil, entre 3,06% e 4,04% das reuniões de equipe foram lideradas por profissionais da eSB. No período, o Nordeste e o Sul foram as regiões que apresentaram maiores (3,71% a 4,88%) e menores proporções (1,21% a 2,48%), respectivamente. No período de 2018 a 2022, houve uma redução do indicador “grau de protagonismo das eSB” no Brasil e nas macrorregiões. Os temas mais frequentes em reuniões sob responsabilidade das eSB foram processo de trabalho (54,71% a 70,64%) e diagnóstico e monitoramento do território (33,49% a 54,48%). As maiores disparidades entre as regiões foram observadas para o indicador “grau de organização das eSB, em relação à discussão de caso e de projeto terapêutico singular”.

**CONCLUSÕES:**

O protagonismo das eSB no processo de trabalho em equipe na APS é incipiente e apresenta disparidades regionais, o que desafia gestores e eSB para o rompimento do isolamento e da falta de integração, visando a oferta de atenção à saúde integral e de qualidade ao usuário do Sistema Único de Saúde (SUS).

## INTRODUÇÃO

A Atenção Primária à Saúde (APS) se configura como o centro articulador do acesso dos usuários ao Sistema Único de Saúde (SUS). A Política Nacional de Atenção Básica define a Estratégia da Saúde da Família (ESF) como modelo de atenção prioritário para a consolidação e a ampliação da cobertura da APS no país^1^, assim como do espaço estratégico para a qualificação da oferta das ações de saúde do SUS^2^. A experiência de trabalho na ESF possibilita o desenvolvimento de ações que visam mudanças na prática de saúde e na autonomia dos sujeitos participantes dessa proposta^3^.

A atuação conjunta, de forma colaborativa, dos profissionais da saúde, é um dos pilares da organização do processo de trabalho proposta pela ESF para a solução dos problemas de saúde. A vinculação da equipe de Saúde Bucal (eSB) na ESF favorece a reorientação do processo de trabalho para um modelo de atenção à saúde^4^, preconizando a articulação das ações, a interação comunicativa dos trabalhadores e a superação do isolamento dos saberes^5^. O cuidado em Saúde Bucal passa a exigir a configuração de uma equipe que se relacione com os usuários e com os demais profissionais, bem como participe da gestão dos serviços para responder às demandas da população, por meio do planejamento de ações individuais e coletivas de promoção, prevenção e recuperação da saúde, num determinado território^4^. A realização de atividades conjuntas nas Unidades Básicas de Saúde (UBS) e no território constitui-se em uma estratégia essencial para o trabalho em equipe, podendo indicar o nível de integração entre os profissionais.

Os desafios para a organização dos processos de trabalho em saúde bucal na APS são persistentes, tais como a fragilidade na integração da eSB com a equipe da ESF e a falta de uma gestão participativa, o que ocasiona insatisfação dos profissionais e usuários^6^. As eSB desenvolvem poucas ações articuladas com as demais equipes na ESF, sendo essa integração considerada incipiente^7,8,9,10^e se recomendando melhorias na participação das eSB no planejamento conjunto das ações desenvolvidas^11,12,7,8^. Conhecer a forma que o processo de trabalho das eSB está sendo desenvolvido pode apontar facilidades e dificuldades, direcionando o planejamento das ações^13^ voltadas para uma atenção integral e de qualidade^14^. Por essa razão, o monitoramento e a avaliação da gestão e atenção em saúde bucal devem ser fomentados no Brasil para ampliar a capacidade avaliativa dos serviços públicos de saúde.

O monitoramento de indicadores gerados a partir de dados obtidos no cotidiano dos serviços de saúde é uma importante estratégia para avaliar o processo de trabalho em saúde. O Sistema de Informação em Saúde para a Atenção Básica (SISAB) disponibiliza os dados registrados pelos profissionais por meio da estratégia e-SUS APS, incluindo as atividades coletivas realizadas com a população e com a equipe. Essas atividades abrangem as reuniões entre equipes e as com instâncias locais de Controle Social^15^. A participação nas reuniões de equipe é uma atribuição comum dos profissionais que atuam na APS, se caracterizando pela discussão conjunta, pelo planejamento e pela avaliação das suas ações, a partir da utilização dos dados disponíveis^1^. Essas reuniões favorecem a integralidade das ações de saúde, contribuindo para a organização do serviço e para um melhor conhecimento das necessidades da população adscrita^1^. Reuniões entre usuários, profissionais e gestores promovem maior proximidade entre esses atores sociais e se conformam como espaços privilegiados para o exercício da democracia participativa e do controle social sobre os serviços de saúde^16^. Nessa perspectiva, seis indicadores foram construídos e validados para medir, de forma inédita, o protagonismo das eSB no processo de trabalho em equipe, a partir de dados disponibilizados pelo SISAB^2^.

Considerando as evidências das desigualdades regionais na organização do processo de trabalho das eSB na APS^14^, justifica-se a comparação entre as macrorregiões brasileiras para revelar os aspectos da atuação e o protagonismo das eSB no cotidiano do trabalho multiprofissional na APS^2^. A análise que considera os contextos regionais poderá subsidiar a tomada de decisão, tendo como referência o princípio da equidade, comprometida com um cuidado integral e a qualificação da atenção aos usuários, útil e oportuno para o novo ciclo de Políticas de Saúde Bucal, iniciado em 2023^2^. Nesse contexto, o objetivo deste estudo foi avaliar e comparar o protagonismo das eSB no processo de trabalho em equipe na APS ao longo de cinco anos (de 2018 a 2022) e estimar a magnitude das disparidades entre as regiões brasileiras para o ano de 2022.

## MÉTODO

Trata-se de um estudo ecológico de âmbito nacional, que utilizou os dados secundários públicos extraídos do SISAB em janeiro de 2023. Os indicadores analisados foram: grau de protagonismo das eSB nas reuniões de equipe (IND1); grau de organização das eSB em relação ao processo de trabalho da equipe (IND2); grau de organização das eSB em relação às questões administrativas/funcionamento (IND3); grau de organização das eSB em relação ao diagnóstico e monitoramento do território (IND4); grau de organização das eSB em relação à discussão de caso e de projeto terapêutico singular (IND5); e grau de organização das eSB em relação à educação permanente (IND6). Estes indicadores foram validados por um comitê de juízes, e a sua mensurabilidade foi testada utilizando dados do ano de 2020^2^. Eles compõem a subdimensão “Processo de Trabalho da Equipe de Saúde Bucal” da dimensão Gestão em Saúde Bucal, da Matriz de Indicadores para Monitoramento e Avaliação da Qualidade dos Serviços de Saúde Bucal^2^.

Os dados utilizados para cálculo dos indicadores são gerados a partir do registro na Ficha de Atividade Coletiva nas aplicações de Coleta de Dados Simplificada (CDS) de modo *online* ou *offline*, no aplicativo e-SUS APS Atividade Coletiva (em dispositivos *Android*^®^) ou por meio de sistemas próprios que alimentam o SISAB. As variáveis utilizadas para obtenção dos indicadores se referem às ações coletivas para a organização da equipe, que incluem reunião de equipe, reunião com outras equipes de saúde, e reunião intersetorial/Conselho Local de Saúde (CLS)/Controle social. Os temas das reuniões podem ser um ou mais dos seguintes: questões administrativas/funcionamento; processo de trabalho; diagnóstico do território/monitoramento do território; planejamento/monitoramento das ações da equipe; e/ou discussão de caso/projeto terapêutico singular e educação permanente. Os dados do profissional, do Cartão Nacional de Saúde (CNS) e da Classificação Brasileira de Ocupações (CBO), mediadores da atividade coletiva, devem ser informados em cada registro e são obrigatórios^15^.

A obtenção dos dados do numerador e do denominador de cada indicador foi realizada por consulta ao “Relatório de Atividade Coletiva na Atenção Básica” do SISAB, sendo extraídos em nível nacional e para as cinco macrorregiões brasileiras para cada ano entre 2018 e 2022.

O grau de protagonismo das eSB (IND1) se refere à proporção de reuniões sob responsabilidade dos profissionais da eSB e é calculado pela razão entre o número de reuniões de equipe, reuniões com outras equipes de saúde, reuniões Intersetorial/Conselho Local de Saúde/Controle Social, sob responsabilidade de profissional da ESB em determinado local e período, e o número de reuniões realizadas no mesmo local e período^2^. As outras equipes (Eq.) são: Eq. de Saúde da Família (ESF); Eq. de Agente Comunitário de Saúde (EACS); Núcleo de Apoio à Saúde da Família (NASF); Eq. da Atenção Básica (EAB), Eq. Consultório na Rua (ERC); Eq. AB Prisional (EABp); e Eq. de Atenção Primária (EAP)^15^.

Os demais indicadores medem a proporção das temáticas registradas nas reuniões sob responsabilidade dos profissionais da eSB, sendo estimados pela razão entre o número de reuniões de equipe, reuniões com outras equipes de saúde em que o profissional responsável era membro da eSB em que foi registrada uma das temáticas (questões administrativas/funcionamento, processo de trabalho, diagnóstico do território/monitoramento do território, planejamento/monitoramento das ações da equipe, discussão de caso/projeto terapêutico singular e educação permanente) em determinado local e período; e o número de reuniões de equipe e reuniões com outras equipes, em que o profissional responsável era membro da eSB independentemente do tema registrado, no mesmo local e período^2^. Para extração dos dados, foram selecionados os filtros no SISAB de acordo com o dado a ser obtido, conforme orientações presentes na Figura para obtenção do numerador (NUM) e denominador (DEM) de cada indicador^2^.

**Figura:** Filtros no SISAB para obtenção do numerador (NUN) e denominador (DEM) dos indicadores2

Lista de Siglas: IND: Indicador/NUN: Numerador/DEM: Denominador/Eq: Equipe/SB: Saúde Bucal

^1^ Outras equipes: Eq. de Saúde da Família – ESF, Eq. de Agente Comunitário de Saúde – EACS, Núcleo de Apoio à Saúde da Família – NASF, Eq. da Atenção Básica – EAB, Eq. Consultório da Rua – ERQ, Eq. AB Prisional – EABp e Eq. de Atenção Primária – eAP.

^2^ Profissionais: Agente comunitário de saúde, Agente de combate a endemias, Agente de saúde, Assistente Social, Cirurgião dentista, Educador social, Enfermeiro, Farmacêutico, Fisioterapeuta, Fonoaudiólogo, Médico, Médico veterinário, Nutricionista, Outros prof. de nível médio, Outros prof. de nível superior, Profissional de educação física, Psicólogo, Sanitarista, Técnico e auxiliar de enfermagem, Técnico e auxiliar de saúde bucal, Terapeuta ocupacional, Naturólogo, Musicoterapeuta, Arteterapeuta, Terapeuto Holístico, Recepcionista.

^3^ Temas de reunião: Questões administrativas/funcionamento, Processos de trabalho, Diagnóstico do território/monitoramento do território, Planejamento/monitoramento das ações da equipe, Discussão de caso/projeto terapêutico singular, Educação permanente, Outros (Temas não se enquadram em nenhuma destas temáticas).

^4^ Turnos: Manhã, Tarde, Noite.

O processo de extração gerou duas planilhas, por indicador, no formato *Excel*^®^: uma para o denominador e outra para o denominador, por ano. Em seguida, foi realizada a vinculação dessas bases, considerando as variáveis comuns que indicavam o nível de desagregação nacional ou cada uma das regiões, e os indicadores foram calculados, dividindo-se o numerador pelo denominador e multiplicando por 100 para obtenção dos valores em percentuais.

A análise descritiva dos indicadores foi realizada considerando os valores calculados para o Brasil e de forma desagregada para cada macrorregião ao longo do tempo (2018-2022). Foi estimada a variação percentual anual para cada indicador, para o Brasil e para cada macrorregião pela taxa de variação (Tx. Var.), empregando o seguinte método de cálculo: *[(resultado do indicador no ano posterior ÷ resultado do indicador no ano anterior)-1]×100*. A variação no período completo foi estimada pela média entre as quatro variações obtidas no período. O Índice de Disparidade (ID) foi empregado para estimar e comparar a magnitude das diferenças dos indicadores entre as macrorregiões brasileiras no ano de 2022. Os valores desse índice indicam o desvio médio das proporções observadas em uma macrorregião, em relação à proporção de referência em percentual, ou seja, o espalhamento das proporções em torno do valor de referência. Neste estudo, o índice de disparidade foi calculado considerando a macrorregião com maior proporção de execução das atividades. O cálculo se baseou na abordagem descrita em Pearcy e Keppel^17^, utilizando a seguinte a fórmula: *ID* = ∑[|*ri* – *R*|/*n*] * 100/*R*, onde *ri =* percentual do indicador, *R* = valor de referência e *n* = número de regiões.

## RESULTADOS

Foram observadas baixas proporções de reuniões sob responsabilidade da eSB ao longo do tempo (Tabela 1). No Brasil, os valores variaram entre 3,06% e 4,04%. Maiores (3,71% a 4,88%) e menores proporções (1,21% a 2,48%) foram observadas para as regiões Nordeste e Sul, respectivamente, em todo o período analisado. As menores proporções de reuniões sob responsabilidade das eSB no Brasil e macrorregiões foram observadas em 2020, coincidente com o início da pandemia da covid-19. Verifica-se, ainda, que ocorreu uma redução (57%) do número total de reuniões realizadas por todas as equipes da APS no Brasil: de 1.029.090, em 2019, para 594.760, em 2020 (tabela 1). As variações percentuais anuais do indicador grau de protagonismo das eSB revelaram um padrão instável de mudança, com aumento e redução entre os anos e diferenças entre as macrorregiões nessas variações. Observou-se, de modo consistente, uma redução ≥ 19.91% para todas as macrorregiões brasileiras em 2020, em relação a 2019. Observou-se também que essa redução foi seguida de uma taxa de variação positiva em todas as macrorregiões no período seguinte (de 2020 para 2021), mantendo-se estável ou com pequenas variações negativas no último ano analisado (Tabela 2). As diferenças do indicador “grau de protagonismo da eSB” entre as regiões foram semelhantes ao longo do período, demonstrando menores e maiores valores consistentemente nas regiões Sul e Nordeste.

**Tabela 1:** Descrição dos indicadores de processo de trabalho em equipe. Brasil e macrorregiões brasileiras (2018-2022).

Região	2018			2019			2020			2021			2022		
				
	N	D	IND (%)	N	D	IND (%)	N	D	IND (%)	N	D	IND (%)	N	D	IND (%)
IND1 - Grau de protagonismo das eSB nas reuniões de equipe															
Brasil	35830	886283	4,04	41531	1029090	4,04	18223	594760	3,06	19174	577055	3,32	23.070	694.807	3,32
Centro-oeste	2522	59647	4,23	2843	67302	4,22	1320	39057	3,38	1467	37461	3,92	1.896	49.902	3,80
Nordeste	19441	410953	4,73	24442	500704	4,88	9907	266898	3,71	9750	246386	3,96	10.344	259.148	3,99
Norte	2243	70715	3,17	3450	98437	3,50	1524	64403	2,37	1737	63213	2,75	1.859	70.801	2,63
Sudeste	8738	228408	3,83	8256	252250	3,27	4776	167061	2,86	5438	170508	3,19	7.638	230.051	3,32
Sul	2886	116560	2,48	2540	110397	2,30	696	57341	1,21	782	59487	1,31	1.333	84.905	1,57
IND2 - Grau de organização das eSB em relação ao processo de trabalho da equipe															
Brasil	17842	32613	54,71	21612	37845	57,11	10799	17332	62,31	11132	17843	62,39	13129	21217	61,88
Centro-oeste	1480	2387	62,00	1798	2665	67,47	832	1249	66,61	1000	1416	70,62	1263	1788	70,64
Nordeste	9097	17556	51,82	12428	22336	55,64	5980	9499	62,95	5641	9142	61,70	5767	9652	59,75
Norte	1332	2108	63,19	1924	3170	60,69	969	1460	66,37	1009	1594	63,30	1226	1741	70,42
Sudeste	4316	8025	53,78	4136	7519	55,01	2592	4499	57,61	3063	5021	61,00	4122	6861	60,08
Sul	1617	2537	63,74	1326	2155	61,53	426	625	68,16	413	670	61,64	685	1175	58,30
IND3 - Grau de organização das eSB em relação às questões administrativas/funcionamento															
Brasil	13301	32613	40,78	16765	37845	44,30	8051	17332	46,45	8117	17843	45,49	9048	21217	42,65
Centro-oeste	1078	2387	45,16	1438	2665	53,96	762	1249	61,01	811	1416	57,27	1069	1788	59,79
Nordeste	6852	17556	39,03	9933	22336	44,47	4630	9499	48,74	4351	9142	47,59	4197	9652	43,48
Norte	946	2108	44,88	1285	3170	40,54	632	1460	43,29	694	1594	43,54	791	1741	45,43
Sudeste	3109	8025	38,74	2941	7519	39,11	1688	4499	37,52	1912	5021	38,08	2413	6861	35,17
Sul	1316	2537	51,87	1168	2155	54,20	339	625	54,24	349	670	52,09	578	1175	49,19
IND4 - Grau de organização das eSB em relação ao diagnóstico e monitoramento do território															
Brasil	13335	32613	40,89	16332	37845	43,15	7429	17332	42,86	7524	17843	42,17	9469	21217	44,63
Centro-oeste	833	2387	34,90	1151	2665	43,19	479	1249	38,35	571	1416	40,32	820	1788	45,86
Nordeste	7394	17556	42,12	10057	22336	45,03	4550	9499	47,90	4294	9142	46,97	4371	9652	45,29
Norte	849	2108	40,28	1189	3170	37,51	489	1460	33,49	638	1594	40,03	716	1741	41,13
Sudeste	3112	8025	38,78	2987	7519	39,73	1615	4499	35,90	1665	5021	33,16	2923	6861	42,60
Sul	1147	2537	45,21	948	2155	43,99	296	625	47,36	356	670	53,13	639	1175	54,38
IND5 - Grau de organização das eSB em relação à discussão de caso e de projeto terapêutico singular															
Brasil	3606	32613	11,06	4182	37845	11,05	1694	17332	9,77	1675	17843	9,39	2710	21217	12,77
Centro-oeste	317	2387	13,28	384	2665	14,41	156	1249	12,49	188	1416	13,28	287	1788	16,05
Nordeste	1168	17556	6,65	1527	22336	6,84	621	9499	6,54	498	9142	5,45	564	9652	5,84
Norte	192	2108	9,11	232	3170	7,32	114	1460	7,81	98	1594	6,15	145	1741	8,33
Sudeste	1353	8025	16,86	1482	7519	19,71	667	4499	14,83	724	5021	14,42	1486	6861	21,66
Sul	576	2537	22,70	557	2155	25,85	136	625	21,76	167	670	24,93	228	1175	19,40
IND6 - Grau de organização das eSB em relação à educação permanente															
Brasil	6437	32613	19,74	7352	37845	19,43	3501	17332	20,20	3523	17843	19,74	4445	21217	20,95
Centro-oeste	463	2387	19,40	504	2665	18,91	226	1249	18,09	286	1416	20,20	313	1788	17,51
Nordeste	2882	17556	16,42	3526	22336	15,79	1395	9499	14,69	1374	9142	15,03	1585	9652	16,42
Norte	432	2108	20,49	758	3170	23,91	372	1460	25,48	342	1594	21,46	430	1741	24,70
Sudeste	2081	8025	25,93	2056	7519	27,34	1355	4499	30,12	1386	5021	27,60	1809	6861	26,37
Sul	579	2537	22,82	508	2155	23,57	153	625	24,48	135	670	20,15	308	1175	26,21

N: Numerador; D: Denominador; IND: Indicador

**Tabela 2:** Taxa de variação (Tx. Var.) dos indicadores de processo de trabalho em equipe. Brasil e macrorregiões brasileiras (2018-2022) (%)

Brasil/Regiões	Tx. Var.	Tx. Var.	Tx. Var.	Tx. Var.	Média Tx. Var.
				
	2018–2019	2019–2020	2020–2021	2021–2022	2018–2022
IND1 - Grau de protagonismo das eSB nas reuniões de equipe					
Brasil	0,00	-24,26	8,50	0,00	-3,94
Centro-Oeste	-0,24	-19,91	15,98	-3,06	-1,81
Nordeste	3,17	-23,98	6,74	0,76	-3,33
Norte	10,41	-32,29	16,03	-4,36	-2,55
Sudeste	-14,62	-12,54	11,54	4,08	-2,89
Sul	-7,26	-47,39	8,26	19,85	-6,64
IND2 - Grau de organização das eSB em relação ao processo de trabalho da equipe					
Brasil	4,39	9,11	0,13	-0,82	3,20
Centro-Oeste	8,82	-1,27	6,02	0,03	3,40
Nordeste	7,37	13,14	-1,99	-3,16	3,84
Norte	-3,96	9,36	-4,63	11,25	3,01
Sudeste	2,29	4,73	5,88	-1,51	2,85
Sul	-3,47	10,78	-9,57	-5,42	-1,92
IND3 - Grau de organização das eSB em relação às questões administrativas/funcionamento					
Brasil	8,63	4,85	-2,07	-6,24	1,29
Centro-Oeste	19,49	13,07	-6,13	4,40	7,71
Nordeste	13,94	9,60	-2,36	-8,64	3,14
Norte	-9,67	6,78	0,58	4,34	0,51
Sudeste	0,96	-4,07	1,49	-7,64	-2,32
Sul	4,49	0,07	-3,96	-5,57	-1,24
IND4 - Grau de organização das eSB em relação ao diagnóstico e monitoramento do território					
Brasil	5,53	-0,67	-1,61	5,83	2,27
Centro-Oeste	23,75	-11,21	5,14	13,74	7,86
Nordeste	6,91	6,37	-1,94	-3,58	1,94
Norte	-6,88	-10,72	19,53	2,75	1,17
Sudeste	2,45	-9,64	-7,63	28,47	3,41
Sul	-2,70	7,66	12,18	2,35	4,87
IND5 - Grau de organização das eSB em relação à discussão de caso e de projeto terapêutico singular					
Brasil	-0,09	-11,58	-3,89	36,00	5,11
Centro-Oeste	8,51	-13,32	6,33	20,86	5,60
Nordeste	2,86	-4,39	-16,67	7,16	-2,76
Norte	-19,65	6,69	-21,25	35,45	0,31
Sudeste	16,90	-24,76	-2,76	50,21	9,90
Sul	13,88	-15,82	14,57	-22,18	-2,39
IND6 - Grau de organização das eSB em relação à educação permanente					
Brasil	-1,57	3,96	-2,28	6,13	1,56
Centro-Oeste	-2,53	-4,34	11,66	-13,32	-2,13
Nordeste	-3,84	-6,97	2,31	9,25	0,19
Norte	16,69	6,57	-15,78	15,10	5,65
Sudeste	5,44	10,17	-8,37	-4,46	0,70
Sul	3,29	3,86	-17,69	30,07	4,88

No Brasil, os temas mais frequentemente pautados nas reuniões de equipe foram, ao longo do período, processo de trabalho (54,71% a 70,64%), diagnóstico e monitoramento do território (33,49% a 54,48%) e questões administrativas/funcionamento (35,17% a 61,01%). Os temas menos frequentes foram educação permanente (14,69% a 30,12%) e discussão de caso/projeto terapêutico singular (5,45% a 25,85%) (Tabela 1). Essa distribuição se repetiu ao considerar cada macrorregião separadamente, com maior frequência de discussão do tema processo de trabalho da equipe em todas as macrorregiões.

A Tabela 2 evidencia a pequena variação dos indicadores ao longo do tempo em todas as macrorregiões, assim como as diferenças entre elas nas proporções observadas. As variações percentuais anuais foram instáveis, em magnitude, entre as macrorregiões e ao longo do tempo. As médias dessas variações no período completo (2018–2022) foram positivas para a maioria dos indicadores no Brasil e na maioria das macrorregiões, sinalizando um padrão de aumento nas proporções.

O IND4 – “Diagnóstico e monitoramento do território” apresentou as maiores taxas de variação positiva em dois momentos, em 2018-2019 (23,75%), na região Centro-Oeste, e em 2020-2021 (19,53%), na região Norte. O IND5 – “Discussão de caso e de projeto terapêutico singular” apresentou em todos os períodos as maiores variações negativas, em duas macrorregiões específicas, sendo no período 2018-2019 (-19,65%) e 2020-2021 (-21,25%) na região Norte, e 2019-2020 (-15,82%) e 2021-2022 (-22,18%) na região Sul. Observou-se uma grande oscilação do IND5 na região Sudeste, que apresentou variação positiva no período 2018-2019 (16,90%), depois variação negativa em 2019-2020 (-24,76%), e novamente variação negativa em 2020-2021 (-2,76%) e, em seguida, a maior variação encontrada para todos os indicadores estudados em 2021-2022 (50,21%), resultando em uma média positiva de (9,90%) para o período 2018-2019.

No ano de 2022, observou-se diferentes magnitudes de disparidades entre as regiões, sendo os maiores valores observados para os indicadores IND 5 – “Grau de organização das eSB em relação à discussão de caso e de projeto terapêutico singular” (ID = 34,2), IND1 – “Grau de protagonismo das eSB nas reuniões de equipe” (ID = 23,3), e IND3 – “Grau de organização das eSB em relação às questões administrativas/funcionamento” (ID = 22,0) (Tabela 3).

**Tabela 3:** Índice de Disparidade (ID) entre as regiões geográficas no grau de protagonismo e na organização das eSB em relação aos temas das reuniões de equipes, Brasil, 2022.

Brasil/Regiões	Indicador 2022 (%)	Índice de Disparidade (ID)
IND1 - Grau de protagonismo das eSB nas reuniões de equipe		
Brasil	3,32	23,3
Centro-Oeste	3,80	
Nordeste	3,99*	
Norte	2,63	
Sudeste	3,32	
Sul	1,57	
IND2 - Grau de organização das eSB em relação ao processo de trabalho da equipe		
Brasil	61,88	9,4
Centro-Oeste	70,64	
Nordeste	59,75	
Norte	70,42*	
Sudeste	60,08	
Sul	58,30	
IND3 - Grau de organização das eSB em relação às questões administrativas/funcionamento		
Brasil	42,65	22
Centro-Oeste	59,79*	
Nordeste	43,48	
Norte	45,43	
Sudeste	35,17	
Sul	49,19	
IND4 - Grau de organização das eSB em relação ao diagnóstico e monitoramento do território		
Brasil	44,63	15,7
Centro-Oeste	45,86	
Nordeste	45,29	
Norte	41,13	
Sudeste	42,60	
Sul	54,38*	
IND5 - Grau de organização das eSB em relação à discussão de caso e de projeto terapêutico singular		
Brasil	12,77	34,2
Centro-Oeste	16,05	
Nordeste	5,84	
Norte	8,33	
Sudeste	21,66*	
IND6 - Grau de organização das eSB em relação à educação permanente		
Brasil	20,95	15,7
Centro-Oeste	17,51	
Nordeste	16,42	
Norte	24,70	
Sudeste	26,37*	
Sul	26,21	

*Regiões com a maior proporção no resultado dos indicadores, utilizadas como valores de referências para o cálculo do índice de disparidade.

Destaca-se que a região Nordeste apresentou o maior valor para o IND1 – “Grau de protagonismo das eSB nas reuniões de equipe” e os menores valores para IND 5 – “Grau de organização das eSB em relação à discussão de caso e de projeto terapêutico singular” e para IND3 –“GRAU de organização das eSB em relação às questões administrativas/funcionamento”.

O inverso foi observado para a região Sul, com menor valor para IND1 – “Grau de protagonismo das eSB nas reuniões de equipe” e valores maiores para os IND3 e IND5. A menor magnitude de disparidade entre as macrorregiões foi observada para o IND2 – “Grau de organização das eSB em relação ao processo de trabalho da equipe” (ID = 9,4) (Tabela 3).

## DISCUSSÃO

Esse trabalho avaliou, de maneira inédita, o protagonismo da eSB no processo de trabalho em equipe na APS, no Brasil e suas macrorregiões, por meio de indicadores elaborados a partir de dados do SISAB. Os resultados mostraram uma pequena proporção de reuniões sobre responsabilidade da eSB e parecem indicar as dificuldades na cogestão do processo de trabalho em saúde na APS. Prática colaborativa, comunicação entre as equipes e o compartilhamento de poder, visando a superação da fragmentação, da atuação profissional isolada e das relações hierárquicas de trabalho^5^ são desafios enfrentados nos serviços de saúde. O estímulo ao protagonismo dos trabalhadores de saúde é um dos princípios da Política Nacional de Humanização do SUS^18^, que visa a participação das equipes nos processos decisórios do trabalho, a fim de consolidar o saber que constroem no seu cotidiano^19^.

As reuniões de equipe são espaços para o fortalecimento do protagonismo dos profissionais, tendo em vista que a sua realização periódica é uma estratégia de aproximação, integração e planejamento da equipe^20^. Como as reuniões são o recurso mais utilizado para propiciar a comunicação entre os profissionais e os usuários, bem como para promover o trabalho em equipe, o baixo protagonismo parece ser reflexo das práticas profissionais das eSB ainda serem marcadas pelo isolamento e pela pouca interação e participação nos processos coletivos de gestão do trabalho e gestão participativa na APS. Por outro lado, podem também refletir problemas de ordem estrutural e de interação para a realização das reuniões, tais como: ausência de espaço físico, modelo de gestão e atenção à saúde bucal vigente; existência de relações conflituosas e distantes; pouca disponibilidade de tempo por parte dos profissionais e pouca valorização institucional desse tipo de atividade^5;20^; inexistência e/ou pequena visibilidade dos conselhos locais de saúde nos territórios; e também uma pequena apropriação desses espaços por parte das eSB.

Esses achados também podem estar relacionados à diferença de cobertura populacional entre as eSB e eSF. Em 2020, a cobertura de eSF no Brasil era de 63,62%, enquanto a de eSB era de 44,95%. Entre as macrorregiões, são observadas diferenças nas coberturas dessas equipes, com disparidades entre as regiões, sendo de 82,33% e 69,56% no Nordeste para eSF e eSB, respectivamente, e 50,99% e 30,09%, respectivamente, no Sudeste^21^. Essa desproporção pode aprofundar as barreiras para o seu protagonismo nas reuniões.

Houve instabilidade na variação temporal da participação das eSB como responsáveis pelas reuniões, demonstrando não haver um padrão de atuação da equipe. A variação negativa observada do ano de 2019 para o ano 2020, em todas as regiões, corresponde ao período em que o processo de trabalho no âmbito da APS, as ações e os serviços foram redefinidos, com significativa redução de atividades coletivas presenciais, visando a contenção da transmissão do Coronavírus^22^. Para avanços no trabalho em saúde bucal na ESF, faz-se necessário que a gestão favoreça os profissionais para buscarem habilidades e competências e que tenham atitudes para proporem ações de intervenção^10^, a fim de promover mudanças positivas no serviço de saúde. Um estudo que analisou a atuação do apoio institucional em relação à participação das eSB em ações de monitoramento, reunião de equipe e organização de processo de trabalho na APS identificou que houve associação positiva das ações realizadas pelo apoiador institucional e as ações não clínicas das eSB no Brasil^23^.

Apesar da baixa proporção das reuniões sob coordenação das eSB, os temas registrados abrangem, com diferentes proporções, aspectos relacionados à organização do processo de trabalho em saúde na UBS e no território. Os temas mais frequentes foram processos de trabalho da equipe e aspectos administrativos e de funcionamento, tanto da UBS quanto de diagnóstico e monitoramento de ações no território. A temática processo de trabalho apresentou proporções mais elevadas em todos os anos do período estudado, tendo médias positivas nas taxas de variação em todas as regiões, com exceção da região Sul. Além disso, essa temática demonstrou o menor índice de disparidade, o que pode sugerir uma possível tendência ao longo do tempo.

A maior frequência desses temas pode indicar maior demanda de realização ou atividades mais consolidadas no trabalho em equipe. Por outro lado, ao se dedicarem mais a esses temas, as equipes podem estar deixando de discutir outras temáticas igualmente importantes para organização do processo de trabalho. As maiores disparidades foram observadas nos indicadores relacionados à temática discussão de caso/projeto terapêutico singular (IND5) e no grau de protagonismo das eSB (IND1). O IND5 também apresentou maiores taxas de variação negativa no período estudado, e o IND1 teve variação negativa em todas as regiões na média dos anos do estudo (2018–2022). Esses mesmos indicadores foram os que apresentaram menores proporções nos valores calculados ao longo do tempo.

As temáticas discussão de casos/projeto terapêutico singular e de educação permanente foram menos frequentes, indicando que o cuidado ao usuário pode estar segmentado e direcionado para tratamentos que não considerem as possibilidades terapêuticas no próprio território^24^. Ao construir o projeto terapêutico de forma compartilhada com a equipe, é possível encontrar respostas às necessidades de saúde bucal de maneira mais resolutiva^25^e a ampliação de vínculo com os usuários^7^.

A Educação Permanente em Saúde (EPS) é uma estratégia que busca a qualificação dos trabalhadores, favorecendo a atuação em equipe, a gestão participativa e a corresponsabilização nos processos de ensino-aprendizagem para o alcance dos objetivos estratégicos do SUS^26^. A baixa proporção de reuniões com a temática EPS pode estar relacionada com a sobrecarga de trabalho, a falta de planejamento para realização das iniciativas de EPS, a não valorização dessas iniciativas pela gestão, entre outras^29^.

Nossos achados corroboram que as diferenças regionais e a heterogeneidade do processo de trabalho eSB no Brasil se mantém^14,30^, indicando que o desempenho dos serviços de saúde está subordinado aos determinantes contextuais. Um estudo de base nacional mostrou que as regiões Sudeste, Sul e Nordeste tiveram as maiores frequências de eSB com melhor desempenho no processo de trabalho quanto ao uso de instrumentos utilizados para planejamento das ações, ações de promoção da saúde e atenção integral. O planejamento das ações se mostrou também como um desafio, com menores proporções de eSB de tipos I e II consolidadas e a maior disparidade entre as regiões brasileiras^14^. Identificar e compreender as diferenças socioeconômicas e de organização dos serviços de saúde podem ajudar os gestores e profissionais a atuarem na redução das disparidades loco-regionais da organização do processo de trabalho das eSB.

Este estudo estimou os indicadores agregados por macrorregiões brasileiras, com uma abordagem exploratória e descritiva, impossibilitando analisar a variabilidade entre os municípios em uma mesma região. Optou-se por esse nível de desagregação, em função do percentual de municípios sem registro de reuniões no período, em todas as regiões, independentemente do profissional responsável, correspondente ao denominador do IND1: Norte (41,5% a 54,4%), Nordeste (6,5% a 25,8%), Sudeste (22,1% a 39,2%), Centro-Oeste (29,1% a 57.4%) e Sul (0 a 39,7%).

Somou-se a esse aspecto o fato de que muitos dos municípios, em todas as regiões, apresentaram ausência de registro de reuniões sob responsabilidade da eSB. Para estes casos se assumiu no numerador, em prol da análise do IND1, que a eSB não se responsabilizou por nenhuma reunião no período analisado. Os percentuais de municípios com nenhuma reunião sob responsabilidade da eSB no período foram: Norte (39,1% a 16,9%), Nordeste (55,7% a 63,2%), Sudeste (52,5% a 62,9%), Centro-Oeste (39,9% a 61,1%) e Sul (51,5% a 83,8%).

Os IND2 a IND6 foram analisados tendo como universo o total de reuniões sob responsabilidade da eSB em cada macrorregião, registradas em municípios com presença de, pelo menos, uma no período, o que correspondeu aos seguintes percentuais: Norte (6,4 a 16,2%), Nordeste (13,2% a 37,5%), Sudeste (6,8% a 16,6%), Centro Oeste (1,8% a 12,3%) e Sul (6,8% a 24,8%). Eles sinalizam e reforçam a fragilidade do protagonismo das eSB nas reuniões de equipe e com os CLS na APS, mostrando a necessidade de qualificar o registro no sistema e-SUS APS para ressignificar e reafirmar as atividades coletivas como elemento central para o processo de trabalho em saúde.

Alguns estudos indicam que são necessários investimentos em recursos tecnológicos, qualificação e treinamento de profissionais e suporte para qualificar a implantação e uso de sistemas de informação no país, especialmente para a estratégia e-SUS APS^30,31^. Fontes de dados secundários disponibilizados por meio de sistemas de informação de base nacional, como o SISAB, constituem-se em fontes relevantes, dada a sua abrangência e capilaridade, mas a baixa qualidade e a ausência dos registros continuam a ser uma barreira para o seu uso.

Não obstante as limitações indicadas, essa análise exploratória inicial dos indicadores inéditos demonstrou o panorama nacional em relação ao protagonismo e grau de organização das eSB no processo de trabalho em equipe delas no Brasil e nas macrorregiões. Além disso, a utilização e avaliação da qualidade dos dados do SISAB podem contribuir para o seu aprimoramento, se consolidando como uma importante fonte para estudos sobre processos de trabalho das equipes da APS.

Outra limitação deste estudo foi a definição de protagonismo da eSB adotada na construção do indicador, que o considerou apenas quando o profissional responsável pela reunião é o cirurgião-dentista, técnico ou auxiliar em saúde bucal. Essa definição pode não corresponder necessariamente ao nível de protagonismo da eSB na condução das atividades, assim como a sua efetiva participação no cotidiano do processo de trabalho em equipe na APS2. Verifica-se também a impossibilidade, por meio do indicador calculado, de estabelecer comparações do protagonismo entre diferentes categorias profissionais que atuam na APS, uma vez que não se estabelece uma relação entre reuniões sob a responsabilidade de diferentes profissionais. Nesse sentido, a elaboração de novos indicadores e estudos futuros se fazem necessários.

Apesar dessas limitações, considera-se que indicadores elaborados a partir do cotidiano do trabalho das equipes na APS apresentam grande potencial para analisar as variações geográficas e temporais em determinadas regiões, estados e/ou municípios, observando os resultados em conjunto ou separadamente e permitindo analisar as disparidades na gestão do processo de trabalhos das eSB e suas possíveis causas. A investigação desses indicadores pode identificar situações de desigualdade e tendências que demandam ações e estudos específicos, contribuindo para a tomada de decisão dos profissionais e gestores da APS^2^.

O protagonismo das eSB no processo de trabalho em equipe na APS no Brasil ainda é incipiente e apresenta disparidades entre as macrorregiões, desafiando gestores e profissionais para a superação do isolamento e da pouca integração entre as diferentes categorias de trabalhadores, tendo em vista a atenção à saúde integral e de qualidade ao usuário do SUS.
